# Capacity Performance for Full-Duplex Multihop Wireless Networks Using Channel Interference Balancing Allocation Scheme

**DOI:** 10.3390/s22093554

**Published:** 2022-05-07

**Authors:** Aung Thura Phyo Khun, Yuto Lim, Stepan Kucera

**Affiliations:** 1 Graduate School of Advanced Science and Technology, Japan Advanced Institute of Science and Technology, 1-1 Asahidai, Nomi City 923-1292, Ishikawa, Japan; ylim@jaist.ac.jp; 2 Nokia Solutions and Networks GmbH, Werinherstrasse 91, 81541 Munich, Germany; stepan.kucera@nokia.com

**Keywords:** cooperative transmission, interference-aware channel allocation, full-duplex system, multihop wireless network

## Abstract

Full-duplex (FD) communication has been attractive as one of the research interests related to spectrum utilization for wireless networks from the previous evolution of communication systems. Previous studies discuss the realization of the FD system by focusing on self-interference cancellation and transmit power control in low-power wireless network scenarios. Today, capacity maximization is a key challenge in FD multihop wireless networks, in which the multi-channel allocation may lead to imbalance interference power due to the different number of simultaneous transmissions and its group selection that occurred on the same sub-channels. In this paper, we focus on the capacity maximization of the FD system by considering the influence of total interference power on each sub-channel and how to balance by selecting the different number of simultaneous transmissions to form a group that leads to a minimum difference in the total interference power on those sub-channels. Therefore, a channel interference balancing allocation (CIBA) scheme for balancing the total interference power in the multi-channel multihop wireless networks is proposed and further investigated by the idea of cooperative transmission. We also adopt the concept of *interference distance* to overcome the interference balancing problem of the proposed CIBA scheme. Performance evaluation results reveal that the proposed CIBA scheme achieves lesser total interference power and higher achievable capacity than other fixed channel allocation schemes.

## 1. Introduction

Full-duplex (FD) communication has attracted attention as one of the research interests related to spectrum utilization for modern wireless networks from the previous mobile wireless evolution, i.e., the 5G mobile communication system. With FD communication, the wireless nodes can simultaneously transmit and receive in the same frequency band different from the half-duplex (HD) communication, which transmits and receives non-simultaneously [1,2]. Different approaches to FD communication have been studied for various types of wireless networks to realize the potential benefits of the FD system. Recently, the great progress in FD communication shows that it can be realized by mainly focusing on the self-interference cancellation and transmit power control for low-power wireless network scenarios with the resource allocation scheme, medium access control (MAC) protocol, single-frequency hardware specification, and so on [3,4,5]. Through those research studies, the FD system brings the advantages of improving spectral efficiency, doubling the capacity of the transmission, reducing the packet collision because of the hidden terminal problem, and so on. However, ever-increasing demands for wireless communication are still necessary to look into the capacity maximization since the future coming 6G mobile communication system is expected to highly massive scale communication.

Capacity maximization becomes a prominent challenge to figure out the traffic explosion of future wireless communication because of the wireless spectrum resource limitation. Therefore, it is worthy to study FD communication for capacity maximization as one of the visions in the 6G mobile communication system [6]. In the FD system, self-interference (SI) and inter-user interference (IUI) play the main consideration factors compared to the HD system. SI is the residual interfering signal at a node that is caused by the high-power difference between the receiving signal from a transmitter and its own transmitting signal at the same frequency band, which is also called as *in-band full-duplex* [7,8]. Many studies proposed the self-interference cancellation technique to enable the transceiver to receive and transmit at the same time on the same frequency band to mitigate the capacity maximization problem of the FD system [9,10,11,12]. Despite SI, when the wireless medium channel is shared, there is another type of interference, which is the IUI between simultaneous transmissions and affects the capacity gain of the FD wireless system. Goyal et al. [13] stated that the strong IUI signals from a neighbor’s transmissions can lead to the high degradation of overall network capacity. Therefore, to realize the FD system, capacity maximization becomes a significant challenge to consider the tradeoff between the interference power of IUI and the capacity gain of the simultaneous transmission. In this regard, optimal resource management and scheduling were presented as another promising opportuniy to increase the network capacity. In [14], the user-scheduling algorithms are proposed to avoid the high interference effect from the simultaneous transmissions, and then, we conducted the resource allocation, while [15] proposed the sub-channel and power allocation for maximizing the capacity of the simultaneous transmission, which impacts the overall network capacity. However, the existing research with the distributed resource allocation minimizes the transmit power to limit the influence of interference power while maintaining link capacity. Therefore, the tradeoff between minimizing the transmit power and capacity gain becomes a considerable factor for the capacity maximization problem in the FD system.

In this paper, we consider that the multi-channel multihop wireless network uses different transmission channels and allows the wireless nodes equipped with multiple radios [16]. Different from the previous studies of interference suppression schemes, transmit power allocation and user scheduling algorithms, the interference power of the simultaneous transmissions can be mitigated by allocating the nearby transmission into a different channel. In this way, the suppression of the interference power improves the link capacity of the transmissions, which results high overall network capacity. In this paper, the objectives are to study how the channel allocation schemes influence the performance in the multihop wireless networks to maximize the achievable capacity with minimum total interference power. Our three contributions are as follows:

- Numerical analysis on HD and FD systems in a multihop wireless network environment using a multi-channel allocation scheme over the existing HD and FD single-channel wireless networks.
- Elucidation of the different channel allocation schemes which result in different performances of the FD multihop wireless networks over the existing single-channel wireless networks.
- Improving the achievable capacity of the cooperative transmission by sharing the information on balancing the total interference power throughout the entire network among the nodes.

Therefore, the proposed channel interference balancing allocation (CIBA) scheme is introduced to balance the applicability of the multi-channel allocation scheme in terms of interference power in the practical use of FD multihop wireless networks. The simulations reveal that in the FD system, the multi-channel transmission mode outperforms the single-channel transmission mode in the multi-channel multihop wireless network. Unlike other fixed channel allocation schemes in the multi-channel transmission mode, the proposed CIBA scheme mitigates the total interference power and brings a better achievable capacity.

This paper is organized as follows. The related works and motivation of this research are introduced in Section 2. Section 3 presents the system model. In Section 4, we present a novel multi-channel allocation management framework. Section 5 proposes the CIBA scheme to balance the total interference power of multi-channel problems to achieve a better achievable capacity in the multihop wireless networks. Section 6 gives the simulation setup, scenario, and results. Finally, the concluding remarks are drawn in Section 7.

## 2. Related Works

Since the work of Gupta and Kumar [17], plentiful research work has focused on analyzing wireless network capacity under different network topologies and wireless technologies. In prior work, Yao et al. [18] analyzed the system capacity of a novel hybrid model selection method between HD and FD, as X-Duplex, which properly switches between HD and FD based on the instantaneous channel state information (CSI). Many existing research studies focused on considering an FD-capable access point (AP) and HD clients. As an example, the authors in [19] considered the inter-client interference problem in the FD network with HD clients. In their research results, the proposed inter-client interference cancellation method achieves a throughput gain of 1.65 times over the conventional HD networks. Another relevant research work is [20], which presented a hybrid scheduling algorithm for heterogeneous HD-FD infrastructure-based networks to tailor different degrees of centralization at the AP. Chen et al. [21] proposed an asymmetric FD MAC protocol and studied its analytical saturation throughput performance in the presence of the hidden terminal problem. The authors also considered the contention window mechanism with the backoff mechanism to provide a more stable saturation throughput.

In wireless networks, the basic cooperative transmission can be divided into strategies of the single relay and multiple relays. In these relay strategies, it can be further classified into HD and FD relaying modes [22]. Since no research work can support the quality of service requirements in both relaying HD (RHD) and relaying FD (RFD) modes, Cheng et al. [23] proposed an optimal resource allocation scheme in the wireless relay networks with a new parameter of cancellation coefficient to analyze the performance of RFD mode. The authors also derived the resource allocation policies for the hybrid RHD and RFD transmission mode and concluded that the effective capacity of the RFD mode is not just twice as much as that of the RHD mode. In similar research works on RFD mode [24], the authors proposed the co-channel interference estimation and cancellation protocol for unidirectional FD and formulate a time and power resource allocation problem for maximizing the achievable spectral efficiency. Their simulation results reveal that the proposed protocol always outperforms the FD MAC protocol, and the achievable spectral efficiency gain is remarkable under a severe interference environment.

A plethora of effort has been spent within the previous few years to grasp the challenges associated with resource allocation in multihop wireless networks. The problem has been approached from different perspectives, starting from protocol-oriented [21] and analytical solutions. The channel allocation and scheduling algorithm for multi-channel multihop wireless networks are presented as an optimization approach to joint congestion control in [25]. The purpose of maximizing the utility function of new traffic is defined as an optimization problem in which the intensity of incoming traffic, channel loads, interface to channel binding and transmission schedules are jointly optimized by dynamic algorithms. Diakonikolas and Zussman [26] presented analytical results of FD links within the single-channel and multi-channel wireless environment, which are essential for the future development of scheduling, channel allocation and power control algorithms.

Therefore, it is worth studying a research methodology aiming at the design level of practical algorithms based on a solid theoretical background that can be analytically proved to guarantee some level of performance improvements through the simulation approach. This paper aims to provide a simple and clear framework for investigating the performance of multi-channel multihop wireless networks as a function of the number of nodes with different transmission modes, single relay strategies and channel allocation algorithms.

The previous research works on multi-channel allocation schemes mainly considered the HD and FD multihop wireless networks. Therefore, the motivation of this paper is to clear up the capacity maximization problem in the FD multihop wireless network. In addition, the FD system is able to obtain the theoretical doubling capacity with spatial reuse compared with the HD system. Therefore, it is worthy to investigate the effect of applying an interference-based balancing approach on the multi-channel allocation scheme in the FD multihop wireless networks. Additionally, this research work motivates us to look more in depth at how the multi-channel allocation scheme can be applied for future wireless communications.

## 3. System Model

**Figure 1:**
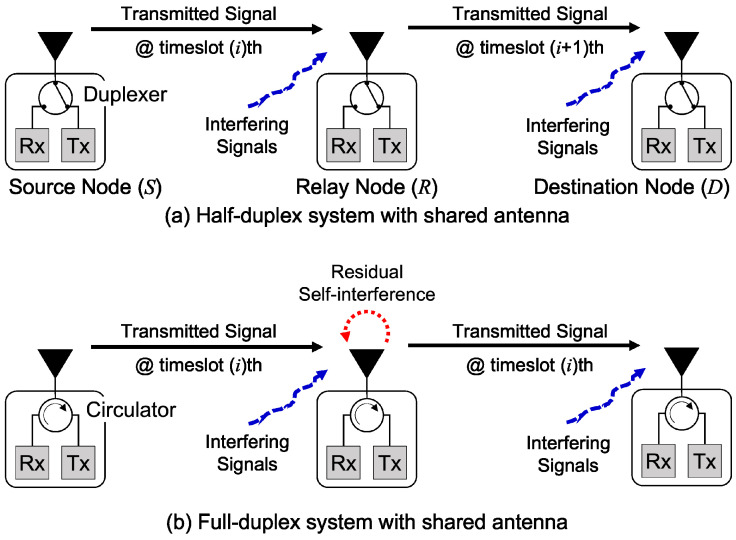
Two types of transmission mode in wireless system.

We consider the multihop wireless network with a single relaying system, in which a source node (*S*) communicates with a destination node (*D*) via a relay node (*R*). Figure 1 illustrates the single relaying system with two types of transmission mode, i.e., an HD system and FD system. In the HD wireless system, the relay node transmits and receives the signal, but only one is allowed to either transmit or receive at the one timeslot. By assuming the self-interference cancellation methods are applied to cancel the self-interference, the relay node transmits and receives the signal simultaneously at a single timeslot with the same frequency in the FD wireless system [23]. In the single relaying system, the relay node can be considered additional relaying strategies (e.g., amplify-and-forward, decode-and-forward, compress-and-forward, etc.) [22,27,28]. However, we assume that no relaying strategies are considered in this research work. In addition, we also neglect the time synchronization problem for simplicity purposes. We simply consider the basic relaying flow (BRF) transmission as the combination of source-relay (S2R) transmission and relay-destination (R2D) transmission just the same as an asymmetric transmission in [20]. Our BRF transmission can apply either HD or FD relaying modes without mixing the nodes of HD and FD systems. Let N={1,…,N} denote the set of nodes. We assume that each BRF transmission can be selected at most M=N/3 in one network topology, where *M* is the total number of BRF transmissions and *N* is the total number of nodes.

Suppose that the whole spectrum is divided into C sub-channels, each with the same bandwidth *B*. Let C={1,…,C} denote the set of sub-channels. We assume that a different sub-channel, *c* where c∈C, is allocated to each source node and/or relay node—S2R transmission and/or R2D transmission, respectively, without loss of generality. Supporting that a source node and/or a relay node is being allocated a single sub-channel, then, its channel allocation indicator is given as $n_{c}$ where n∈N.

### 3.1. Channel Model

The available spectrum is assumed to be organized in C separate sub-channels. For the *c*th sub-channel, the channel gain (decibels) between node *i* and node *j*, where *i*,j∈N depends on the log-distance pathloss model with ITU recommendation [29] is given by

(1) $$PL_{ij}^{c}=PL_{0}^{c}+10\cdot \alpha \cdot \log_{10}\left(\frac{d_{ij}}{d_{0}}\right)+L_{f}-W_{ij}+X_{\sigma }$$

where $PL_{0}^{c}=20\cdot \log_{10}\left(f\right)-28$ and *f* is the frequency of transmission in the unit of MHz, and α is the pathloss exponent is equivalent to 3 in this paper. $d_{0}$ is decorrelation distance, which is 1 m, and $W_{ij}$ is the attenuation. $L_{f}=15+4\left(\kappa -1\right)$ is the floor penetration loss factor with κ floors between the transmitter and the receiver and κ=0. This pathloss model with ITU recommendation is also considering instantaneous fading as a Gaussian random variable ($X_{\sigma }$) with zero mean and standard deviation of σ.

### 3.2. Interference Model

We consider a multihop wireless network with multi-channel transmissions. Most research works use a receiver-oriented interference model, i.e., the Physical Model [17] and Protocol Model [30], to represent an ongoing transmission in the presence of interference. Since co-channel interference is not considered in this paper, we adopt the Physical Model, which is dependent on the interference at the *c*th sub-channel. The signal to interference and noise ratio (SINR) at node *j* from node *i* is

(2) $$SINR_{ij}^{c}=\frac{G_{ij}^{c}\cdot P_{i}^{c}}{\eta_{j}\cdot B+\sum_{k\in K^{c},k\neq i}G_{kj}^{c}\cdot P_{k}^{c}}$$

where the channel gain or received power ratio between wireless nodes *i* and *j* is

(3) $$G_{ij}^{c}=\frac{1}{10^{\left(\frac{PL_{ij}^{c}}{10}\right)}}$$

and $P_{i}^{c}$ is the transmit power of node *i*, η is the noise level, *B* is the channel bandwidth and $K^{c}$ is the set of interfering nodes. In this paper, the interfering node can be decided with the interference model by treating the all other transmitting nodes rather than ongoing transmission as the interfering node in the sub-channel *c*.

The Physical Model assumes that all the wireless nodes access the transmission medium without previously checking if there is a transmission from the neighbor wireless nodes. Then, the effect of interference on the ongoing transmission is evaluated for the channel gain. In this paper, we introduce the concept of *interference distance* to have a better parameter to quantify the additive effect of interfering nodes at different distances from the intended destination node.

**Definition** **1** (Interference Distance). *An interference distance $D_{kj}$ concerning a receiving node j is the total of all distances powered by pathloss exponent, α, over a summation of k simultaneous transmissions, resulting in the signal reception at node j.*

**Lemma** **1.** *Assuming that a node i transmits to another node j at distance $d_{ij}$. Then, other active nodes, K, are performing k simultaneous transmissions, k∈{1,…,K}. Therefore, the interference distance to the intended receiving node j is*

(4) $$D_{kj}=\sum_{k\in K,k\neq i}\frac{1}{d_{kj}^{\alpha }}$$

*where $d_{kj}$ is the distance from interfering node k to the receiving node j.*

**Proof.**  With the assumptions that all the nodes have similar receiver characteristics, such as omnidirectional antenna, same sub-channel, same transmit power and thermal noise, and same physical configuration, the *k* simultaneous transmissions from other nodes will result in $D_{kj}$. However, (2) at the receiving node *j* will result in the successful reception as long as

(5) $$SINR_{ij}\geq \beta$$

where β is an SINR threshold that can be used to ensure the level of link quality in a network. In this paper, β is set to 0 dB. The active node *K* becomes the interfering node at a distance greater than $d_{ij}$; otherwise, they stop their data transmission due to the medium being already successfully contended by the ongoing transmission in between node *i* and node *j*. We use the notation $D_{kj}$ as hereafter called *interference distance* to indicate the total distance of all the interfering nodes to the receiving node *j*.    □

### 3.3. Link Capacity Model

The link rate of transmission is computed according to Shannon’s capacity as in [31]. Therefore, the transmission rate (bit/s) from node *i* to node *j* is

(6) $$R_{ij}=B\cdot \log_{2}\left(1+SINR_{ij}\right)$$

#### Achievable Capacity

In multihop wireless networks, an achievable capacity is dependent on the HD and FD relaying modes. In particular, the HD relaying mode uses two consecutive timeslots to transmit data from a source node to a destination node via a relay node. In contrast, the FD relaying mode uses only one timeslot to complete this data transfer. In other words, we can analogize that the HD and FD relaying modes are performing sequential and simultaneous transmissions, respectively. Suppose we consider the BRF transmission with a source node (*S*), a relay node (*R*), and a destination node (*D*). Thus, the achievable capacity with the sequential transmission or the HD relaying mode can be represented by

(7) $$C_{a}^{HD}=\sum_{m=1}^{M}\left(\frac{{\left(R_{SR}^{m}\right)}^{2}+{\left(R_{RD}^{m}\right)}^{2}}{R_{SR}^{m}+R_{RD}^{m}}\right)$$

where $R_{SR}^{m}$ is the transmission rate from source node *S* to relay node *R* at the *m*th BRF transmission. $R_{RD}^{m}$ is the transmission rate from relay node *R* to destination node *D* at the *m*th BRF transmission. Meanwhile, the achievable capacity with the simultaneous transmission or the FD relaying mode can be expressed by

(8) $$C_{a}^{FD}=\sum_{m=1}^{M}\left(R_{SR}^{m}+R_{RD}^{m}\right)$$

### 3.4. Transmission Mode

In general, the transmission mode can be categorized into two types, i.e., HD transmission mode and FD transmission mode. The HD transmission mode is a form of transmission in which several transmissions are executed sequentially. A similar concept to the time-division multiple access (TDMA), many wireless nodes use the same frequency band by dividing the accessing into different timeslots using a channel access method for shared medium wireless networks. On the other hand, the FD transmission mode is a form of transmission in which several transmitters proceed the transmissions to the other intended receivers during overlapping periods simultaneously.

### 3.5. Cooperative Transmission

In this paper, we consider a simple cooperative relaying network by using a single relay strategy. The relay node does not decode the received signal from the source node, whereas it just relays the signal directly to the destination node depending on the transmission modes. The destination node will receive the same signal from the relay node by assuming the time synchronization issue is neglected. This paper aims to investigate the channel allocation scheme for maximizing the achievable capacity of the FD transmission mode compared to the HD transmission mode without considering any relaying strategies.

## 4. Multi-Channel Allocation Management Framework

**Figure 2:**
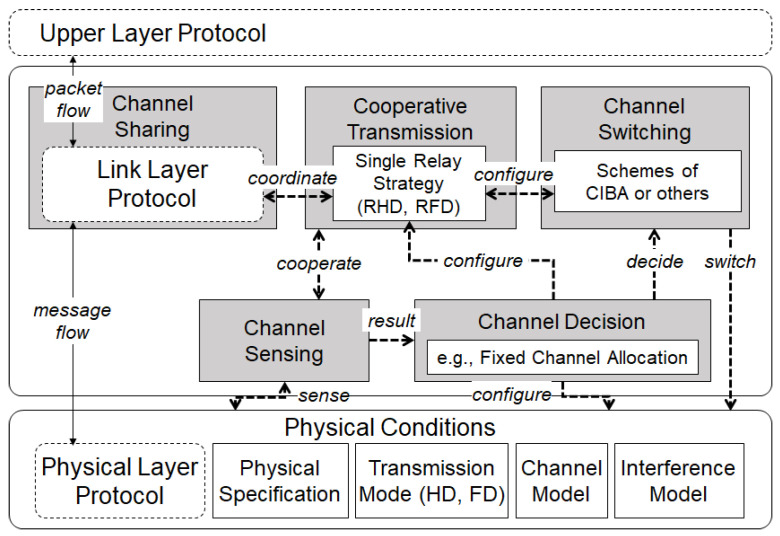
Multi-channel allocation management framework in wireless system environment.

In this section, a framework for multi-channel allocation management is introduced with four components of channel sensing, channel decision, channel switching and channel sharing, and cooperative transmission. Figure 2 illustrates the proposed framework, which is conceptualized with the cooperative transmission manner among wireless nodes, which may operate in either HD or FD transmission modes. The functions and processes of each component are described as follows:

(a) Channel Sensing: wireless node captures and detects the channel conditions of the ongoing transmissions through its transmission mode;
(b) Channel Decision: wireless node finalizes the channel selection based on a channel allocation algorithm and configures the assigned channel for data transfer;
(c) Channel Switching: wireless node switches the pre-assigned channel allocation with an additional advanced algorithm to a new channel allocation by the means of achieving a better cooperative network performance;
(d) Channel Sharing: wireless node shares the channel decision information through a shared wireless medium network with the link layer protocol;
(e) Cooperative Transmission: wireless node performs the transmission cooperation by involving in any BRF transmission through the specified transmission mode.

### 4.1. Channel Allocation Constraints

In this paper, as a preliminary work, we assume that the wireless node can occupy up to two channels. For the relay node, only one channel can be allocated to the HD wireless node in one timeslot; however, two channels can be used for the FD wireless node in one timeslot. All the transmitting nodes can share their channel information with others using one common channel.

### 4.2. Channel Allocation Algorithms

All the transmitting nodes in the BRF transmission are allocated one channel according to a channel allocation algorithm as simplified in Algorithm 1. The algorithm can assign the sub-channel to the source–relay (S2R) transmission and relay–destination (R2D) transmission. Three fixed channel allocation schemes are studied in this paper. The following describes those algorithms.

(a) SR Pairing Channel Allocation (SPCA) Algorithm: SPCA allocates the sub-channel by pairing the transmissions of S2R and R2D. Algorithm 2 shows the channel allocation by SPCA.
(b) SR Transmitting Channel Allocation (STCA) Algorithm: STCA allocates the same sub-channel to the same transmission S2R or transmission R2D of the BRF transmission. In particular, STCA allocates the first sub-channel to the transmission S2R and the second sub-channel to transmission R2D as described in Algorithm 3.
(c) SR Alternating Channel Allocation (SACA) Algorithm: SACA allocates the sub-channel by alternating the sequence of transmissions of S2R and R2D in the BRF transmissions. Algorithm 4 shows how SACA works out the channel allocation.

**Algorithm 1** Channel Allocation Algorithm**Definition:** Assuming the total no. of sub-channels, C = 2, *N* is total no. of nodes, $n_{c}$ is the node that is being assigned as a sub-channel *c*
**Require:** *N***Ensure:** *N*$n_{c}$1: **function**
FCA2:      Set c=C▹*c* is the no. of sub-channels3:      Set M=N/34:      Set m=M▹*m* is the no. of BRF transmissions5:      **for** m←1 to *M* **do**6:        Set $n_{S}^{m}$, $n_{R}^{m}$, $n_{D}^{m}$←*m*▹*n* is the no. of nodes7:      **end for**8:      **function SPCA(*N*, C, $n_{c}$)****9:**      **function STCA(*N*, C, $n_{c}$)****10:**    **function SACA(*N*, C, $n_{c}$)****11:**    **function CIBA(*N*, C, $n_{c}$)****12:**    **Share $\forall n_{c}$ information****13:** **end function**

**Algorithm 2** SR Pairing Channel Allocation Algorithm
1:
**function** SPCA
2:    **while** ∀N is not assigned a sub-channel **do**3:        **for** m←1 to *M* **do**4:           **if** m≤C **then**5:               **for** c←m to C **do**6:                   **if** $S2R^{m}$, $R2D^{m}$ is NULL **then**7:                       Assign $S2R^{m}$, $R2D^{m}$←*c*8:                       Set $n_{S}^{m}$, $n_{R}^{m}$ as $n_{c}$←*c*9:                   **end if**10:               **end for**11:           **else**12:               **for** c←1 to C **do**13:                   **if** $S2R^{m}$, $R2D^{m}$ is NULL **then**14:                       Assign $S2R^{m}$, $R2D^{m}$←*c*15:                       Set $n_{S}^{m}$, $n_{R}^{m}$ as $n_{c}$←*c*16:                   **end if**17:               **end for**18:           **end if**19:        **end for**20:    **end while**21:
**end function**



**Algorithm 3** SR Transmitting Channel Allocation Algorithm
1:
**function** STCA
2:    **while** ∀N is not assigned a sub-channel **do**3:        **for** m←1 to *M* **do**4:           **for** c←1 to C−1 **do**5:               **if** $S2R^{m}$, $R2D^{m}$ is NULL **then**6:                   Assign $S2R^{m}$←*c*7:                   Set $n_{S}^{m}$ as $n_{c}$←*c*8:                   Assign $R2D^{m}$←c+19:                   Set $n_{R}^{m}$ as $n_{c}$←c+110:               **end if**11:           **end for**12:        **end for**13:    **end while**14:
**end function**



**Algorithm 4** SR Alternating Channel Allocation Algorithm
1:
**function** SACA
2:    **while** ∀N is not assigned a sub-channel **do**3:        **for** m←1 to *M* **do**4:           **for** c←1 to C−1 **do**5:               **if** m(mod2)=1 **then**6:                   **if** $S2R^{m}$, $R2D^{m}$ is NULL **then**7:                       Assign $S2R^{m}$←*c*8:                       Set $n_{S}^{m}$ as $n_{c}$←*c*9:                       Assign $R2D^{m}$←c+110:                       Set $n_{R}^{m}$ as $n_{c}$←c+111:                   **end if**12:               **else**13:                   **if** $S2R^{m}$, $R2D^{m}$ is NULL **then**14:                       Assign $S2R^{m}$←c+115:                       Set $n_{S}^{m}$ as $n_{c}$←c+116:                       Assign $R2D^{m}$←*c*17:                       Set $n_{R}^{m}$ as $n_{c}$←*c*18:                   **end if**19:               **end if**20:           **end for**21:        **end for**22:    **end while**23:
**end function**



## 5. Channel Interference Balancing Allocation Scheme

The aim of a channel interference balancing allocation (CIBA) scheme is to switch the existing channel allocation to balance the total interference power of two sub-channels over many simultaneous transmissions. As long as the number of simultaneous transmissions becomes an increase in the multihop wireless network, the total interference power is exponentially increased. By balancing the total interference power among the two sub-channels in the network using a multi-channel allocation scheme, CIBA can improve the achievable capacity of the entire multihop wireless networks. CIBA uses a common channel to compute the interference distance and allocates the sub-channel according to the minimum difference of interference distance between two sub-channels according to the CIBA algorithm. The CIBA algorithm is mainly divided into two main steps balancing the interference and allocating the channel as depicted in Algorithm 5. First, a receiver captures its interference distance from other simultaneous transmissions and shares it with other receivers. Second, the receiver collects other interference distances from other receivers. Third, the receiver balances the total interference power of the two sub-channels. Last, the receiver assigns its sub-channel based on the CIBA algorithm.
**Algorithm 5** Channel Interference Balancing Allocation Algorithm1:**function**CIBA2:    Compute $\forall D_{kR}^{m},D_{kD}^{m}$ of $S2R^{m}$, $R2D^{m}$3:    **while** $min\{diff\left(G_{1},G_{2}\right)\}$ **do**4:        **while** $\forall (D_{kR}^{m},D_{kD}^{m})$ is not assigned into a group **do**5:           Set $G_{1}=rand\left(D_{kR}^{m},D_{kD}^{m}\right)$6:           Set $G_{2}=rand\left(D_{kR}^{m},D_{kD}^{m}\right)$7:        **end while**8:    **end while**9:    **while** ∀N is not assigned a sub-channel **do**10:        **for** m←1 to *M* **do**11:           **if** $S2R^{m}$, $R2D^{m}$ is NULL **then**12:               **if** $S2R^{m}$ is belonging to $G_{1}$ **then**13:                   Assign $S2R^{m}$←*c*14:                   Set $n_{S}^{m}$ as $n_{c}$←*c*15:               **else**16:                   Assign $S2R^{m}$←c+117:                   Set $n_{S}^{m}$ as $n_{c}$←c+118:               **end if**19:               **if** $R2D^{m}$ is belonging to $G_{1}$ **then**20:                   Assign $R2D^{m}$←*c*21:                   Set $n_{S}^{m}$ as $n_{c}$←*c*22:               **else**23:                   Assign $R2D^{m}$←c+124:                   Set $n_{S}^{m}$ as $n_{c}$←c+125:               **end if**26:           **end if**27:        **end for**28:    **end while**29:**end function**

## 6. Numerical Simulations

This section describes the numerical simulation to discuss the performance of the channel allocation schemes with different transmission modes, i.e., HD and FD, in both single-channel and multi-channel multihop wireless network environments.

### 6.1. Simulation Scenarios and Settings

The simulation program is conducted by MATLAB R2021a and is constructed in three parts: (a) the numerical analysis of different transmission modes in a single-channel and multi-channel multihop wireless network; (b) the different performance of fixed channel allocation schemes by considering a multi-channel multihop wireless network with the FD transmission mode; and (c) the numerical performance of the proposed CIBA scheme on balancing the total interference power to improve the achievable capacity. The simulation parameters and settings are shown in Table 1 with the standard specification of IEEE 802.11ac [32]. In the wireless network, the nodes are uniformly distributed over the network coverage area of 100 m × 100 m. As the assumption, all the wireless have the same transmit power and are equipped with FD capability in the FD system. The performance metrics of total interference power and achievable capacity are obtained by averaging 10,000 simulations in the random topology scenarios. The total interference power is the total amount of interference caused in the same channel of the entire network by the neighbour’s simultaneous transmissions. The achievable capacity is the summation of the transmission capacity of every BRF transmission in the network.

### 6.2. Simulation Results and Discussion

The performance analysis of the channel allocation schemes is evaluated as the following.

#### 6.2.1. Performance Analysis of Different Transmission Modes

**Figure 3:**
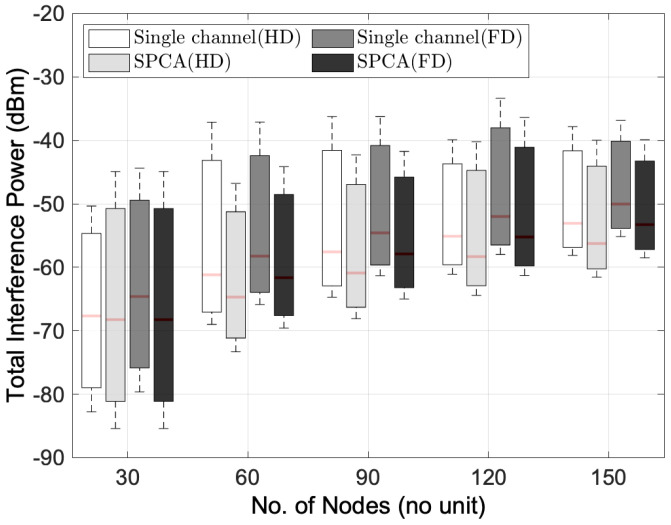
Performance comparison between HD and FD transmission modes for the distribution of total interference power versus the number of nodes.

Figure 3 illustrates the performance comparison between HD and FD transmission modes by considering single-channel allocation and SPCA as a multi-channel allocation algorithm in the multihop wireless network environments. As we can observe from the graph, the HD transmission mode has lower average total interference power than the FD transmission mode in both single-channel and multi-channel multihop wireless network environments. With SPCA as a multi-channel allocation algorithm, the FD transmission mode achieves lower total interference power than the single-channel HD transmission mode. This is because the SPCA is able to assign the channel allocation to reduce the total interference power compared to the HD transmission mode in only one channel even though the sequential transmission is applied. For example, if the number of nodes is 30, when the single-channel HD transmission mode gives −67.66 dBm of total interference power, the multi-channel FD transmission mode can reduce the interference power from −64.60 to −68.24 dBm, which is around 5.6% compared to single-channel FD transmission mode. When the number of nodes is 150, multi-channel FD transmission mode can mitigate the interference to −53.27 dBm from −50.04 dBm which is a 6.4% reduction compared to the single-channel FD transmission mode and around 0.4% reduction compared to −53.07 dBm of the single-channel HD transmission mode. In addition, we can see that the total interference power of both HD and FD transmission modes increases when the node density is high in the wireless networks. Regardless of the number of nodes, although the FD transmission mode brings a higher total interference power than the HD transmission mode in the single-channel multihop wireless network, the FD transmission mode can mitigate the total interference power with the help of a multi-channel allocation algorithm.

**Figure 4:**
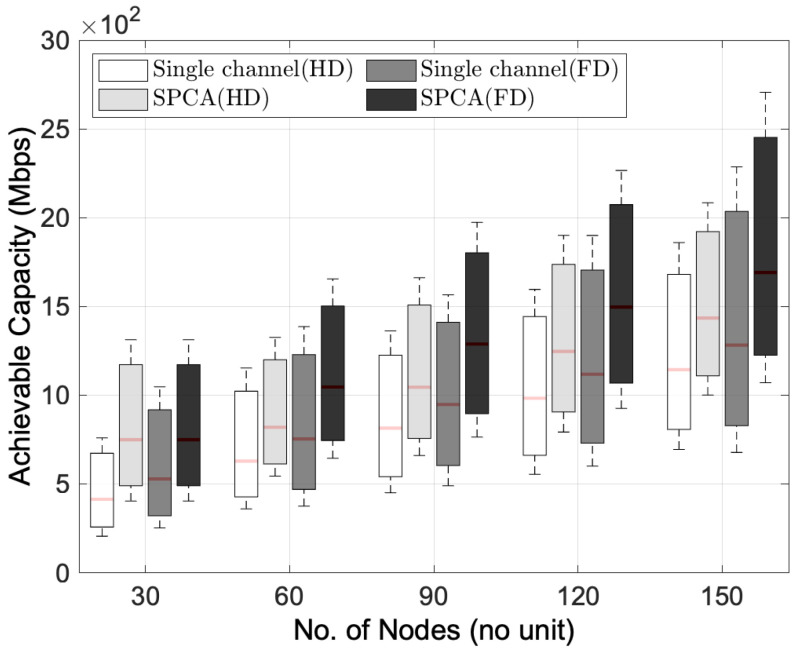
Performance comparison between HD and FD transmission modes for the distribution of achievable capacity versus the number of nodes.

Figure 4 describes the performance in terms of average achievable capacity for HD and FD transmission modes by considering single-channel allocation and SPCA as a multi-channel allocation algorithm in the multihop wireless networks. Regardless of the number of nodes, the multi-channel FD transmission mode with SPCA depicts the superiority of average achievable capacity compared to single-channel FD transmission mode and HD transmission mode. Compared to the single-channel HD transmission mode, the single-channel FD transmission mode still gives a higher achievable capacity, although the total interference power is high. However, since the single-channel FD transmission mode gives higher total interference power than the multi-channel HD transmission mode, the HD transmission mode that applies sequential transmission has a better achievable capacity in the multi-channel multihop wireless network. In addition, another interesting observation in Figure 4 is the improvement of achievable capacity by the multi-channel FD transmission mode. By comparing both the FD transmission mode and HD transmission mode in a single-channel environment, the improvement of achievable capacity is about 1.4 times and 1.8 times, respectively, in the 30-node network topology scenario. In addition, the multi-channel FD transmission mode can give an improvement of 1.3 times and 1.5 times, respectively, compared to the single-channel FD transmission mode and HD transmission mode when the number of nodes is 150 in the network. Although the FD transmission mode brings a higher total interference power than the HD transmission mode in the single-channel multihop wireless network environment, the FD transmission mode can achieve a higher achievable capacity with the help of a multi-channel allocation algorithm regardless of the number of nodes.

#### 6.2.2. Performance Analysis of Fixed Channel Allocation Schemes

**Figure 5:**
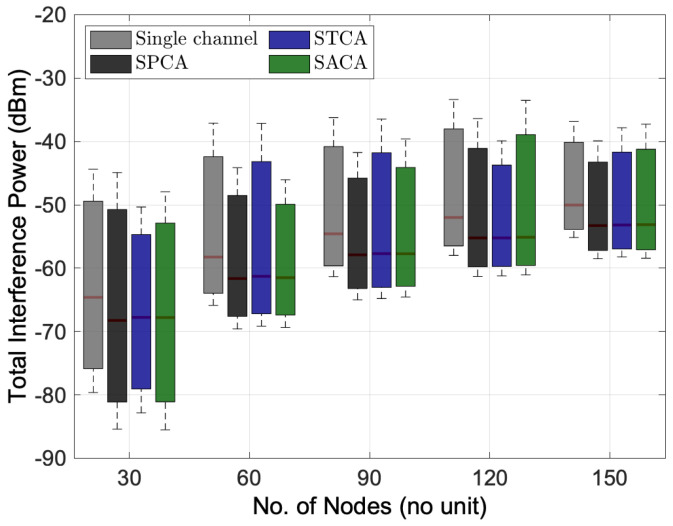
Performance comparison between different fixed channel allocation schemes for the distribution of total interference power versus the number of nodes.

**Figure 6:**
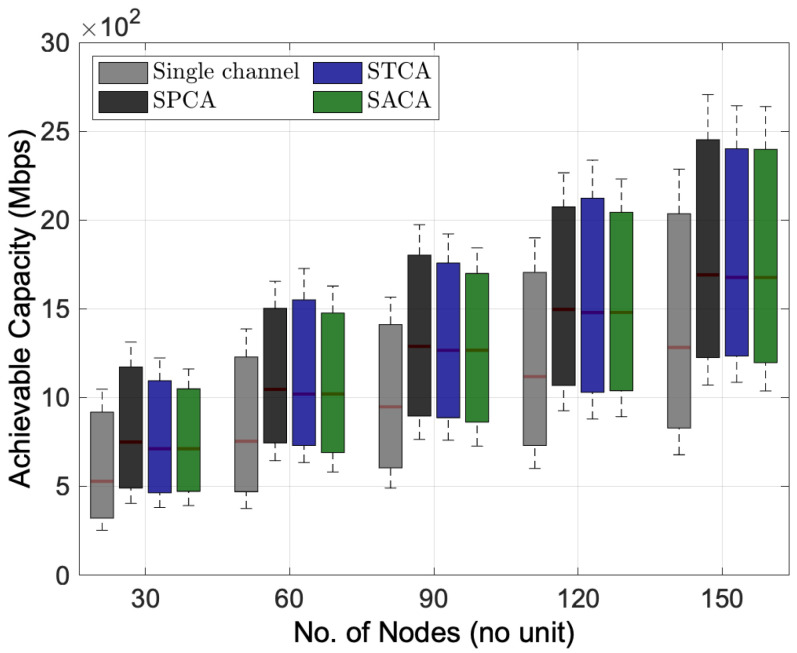
Performance comparison between different fixed channel allocation schemes for the distribution of achievable capacity versus the number of nodes.

Figure 5 and Figure 6 illustrate the performance of different fixed channel allocation schemes by considering multi-channel multihop wireless network environments with FD transmission mode only. As we can see that the three channel allocation algorithms, i.e., SPCA, STCA and SACA, in the multi-channel multihop wireless network give their superiority in terms of total interference power and achievable capacity compared to the single-channel FD transmission mode. Among three different channel allocation algorithms, Figure 6 shows that SPCA brings a higher achievable capacity compared to others, especially, in the low node density wireless networks. Quantitatively, around 5.2% from 712.17 Mbps to 749.08 Mbps when the number of nodes is 30 in the network. With the two sub-channels, by allocating the first sub-channel to the transmission *S2R* and the second sub-channel to the transmission *R2D* of the BRF transmission, and alternating the sequence of transmissions of *S2R* and *R2D* of the BRF transmission in the STCA and SACA, respectively, the simulation results reveal that almost similar interference power and achievable capacity can be obtained by STCA and SACA algorithms.

#### 6.2.3. Performance Analysis of CIBA Scheme

**Figure 7:**
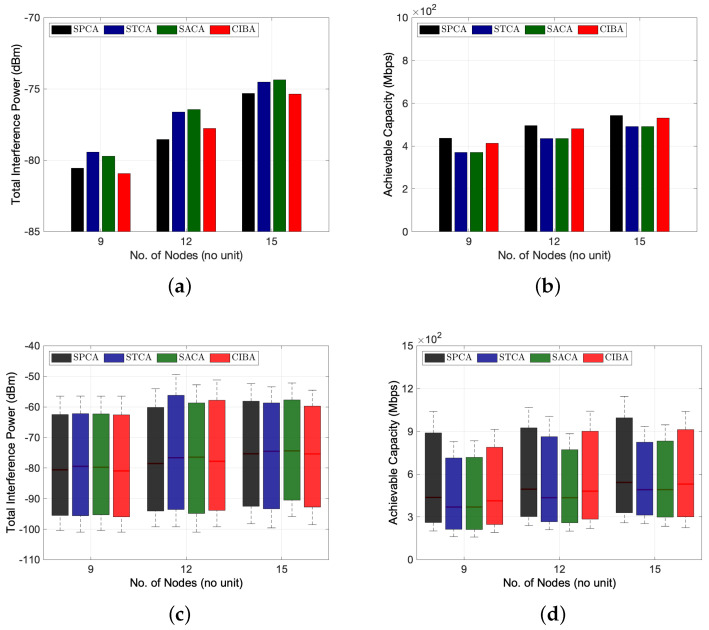
Performance comparison between fixed channel allocation and CIBA algorithms by considering multi-channel FD multihop wireless network environments. (**a**) Performance results for average total interference power versus the number of nodes; (**b**) Performance results for average achievable capacity versus the number of nodes; (**c**) Distribution of total interference power versus the number of nodes; (**d**) Distribution of achievable capacity versus the number of nodes.

Figure 7 depicts the performance of the proposed CIBA scheme compared to the three fixed channel allocation schemes, i.e., SPCA, STCA and SACA, as a function of the number of nodes. The simulation result reveals that the total interference power can be suppressed and achievable capacity can be maximized by balancing the interference power with the multi-channel allocation algorithm in the multi-channel multihop wireless network environments. By balancing the total interference power among the two sub-channels in the multi-channel multihop wireless network, the total interference power of the entire network can be mitigated. Table 2 shows the total interference power comparison of CIBA and the three fixed channel allocation schemes in the 15-node network topology scenario. We can note that CIBA may achieve minimum total interference power in the multi-channel multihop wireless network compared to other fixed channel allocation schemes. In addition, CIBA brings its superiority in terms of achievable capacity as shown in Figure 7b,d. Compared to SICA and SACA, the simulation result reveals that CIBA always gives a higher achievable capacity. The improvement of achievable capacity by CIBA is about 1.2% and 7.9% when the number of nodes is 9 and 15, respectively. However, CIBA may not outperform the SPCA algorithm in some network scenarios, e.g., 15-node network topology. This is because CIBA allocates the channel by mainly focusing on balancing the total interference power of the two sub-channels in the network. CIBA lacks considering the tradeoff between the total interference power and capacity gain of each BRF transmission. Therefore, the possible effort to optimize the performance of CIBA could be balancing the interference power of BRF transmissions instead of two sub-channels in the network. Although SPCA gives a higher achievable capacity than CIBA, CIBA may outperform SPCA by considering the tradeoff between the total interference power and capacity gain of each BRF transmission.

With the assumption of considering only two sub-channels as a preliminary research study for channel allocation in a multi-channel multihop wireless network, we only focus on the design of CIBA with numerical studies in this paper. For considering the time complexity of the channel allocation algorithms, SPCA, STCA and SACA are linear time algorithms and CIBA is a non-linear time algorithm. Through the numerical simulations, CIBA has high time complexity and gives lower total interference power with moderate achievable capacity compared to SPCA, which has low time complexity with higher achievable capacity and moderate total interference power. Due to the time complexity, CIBA is expected to obtain higher achievable capacity compared to SPCA when the number of nodes is very large. In summary, the proposed CIBA brings its superiority in terms of achievable capacity and interference power when the number of nodes increases in the network.

## 7. Conclusions

In this paper, we investigated the performance of the channel allocation schemes and proposed the CIBA scheme by adopting the *interference distance* to solve the minimization problem of balancing total interference power before the sub-channel is allocated to each node in the multi-channel multihop wireless networks. Numerical simulation results reveal that multi-channel allocation schemes in general can enable the FD system to mitigate the entire total interference power and maximize the achievable capacity. Especially when the number of nodes decreases in the network, the FD system can achieve nearly double compared to the HD system. In addition, the proposed CIBA scheme outperforms the fixed channel allocation schemes, especially, SPCA and STCA with higher achievable capacity and lower total interference power. While significant attention has been given to channel allocation in FD multihop wireless networks as we demonstrated, many unknown advantages of FD pose breakthroughs in future 6G mobile communication systems, in particular, the design and optimization of the FD MAC protocol. As our future research work, we will investigate the performance of the CIBA scheme by increasing the number of sub-channels and extend the study of throughput analysis for designing MAC protocol using the Markov Chain approach in the presence of the hidden terminal problem. In addition, the dynamic channel allocation should be further examined when the nodes are moving in the multi-channel multihop wireless networks.

## Figures and Tables

**Table 1:** Simulation parameters and settings.

Parameter	Value
Network coverage size	100 m × 100 m
Number of nodes	9, 12, 15, 30, 60, 90, 120, 150
Transmit power	20 dBm
Propagation model	Log-distance pathloss model (ITU recommendation)
Pathloss exponent (α)	3
Hardware specification	IEEE 802.11ac
Channel bandwidth (*B*)	20 MHz
Noise level (η)	−174 dBm
Number of sub-channels (C)	2
Frequency of sub-channels (*f*)	5.18 and 5.32 GHz
Number of simulations	10,000 times

**Table 2:** Total interference power comparison of CIBA and other fixed channel allocation schemes for 15-node network topology scenario.

Algorithms	Sub-Channel 5.18 GHz	Sub-Channel 5.32 GHz	Entire Network
SPCA	−76.61	−81.21	−75.32
STCA	0.0	−74.51	−74.51
SACA	−81.0	−75.41	−74.36
CIBA	−78.56	−78.19	−75.36

Note: All the values are in the unit of dBm.

## Data Availability

Not applicable.

## Abbreviations

The following abbreviations are used in this manuscript:

*S2R*	Source–relay transmission
*R2D*	Relay–destination transmission
BRF	Basic relay flow transmission
SPCA	Source and relay pairing channel allocation
STCA	Source and relay transmitting channel allocation
SACA	Source and relay alternating channel allocation
CIBA	Channel interference balancing allocation
